# Charge-transfer interaction mediated organogels from 18β-glycyrrhetinic acid appended pyrene

**DOI:** 10.3762/bjoc.9.324

**Published:** 2013-12-16

**Authors:** Jun Hu, Jindan Wu, Qian Wang, Yong Ju

**Affiliations:** 1 Key Laboratory of Bioorganic Phosphorus Chemistry & Chemical Biology, Ministry of Education, Department of Chemistry, Tsinghua University, Beijing, 100084, China,; 2 Department of Chemistry and Biochemistry, University of South Carolina, Columbia, 29208, USA

**Keywords:** charge transfer, glycyrrhetinic acid, organogel, self-assembly

## Abstract

We describe herein the two-component charge-transfer (CT) interaction induced organogel formation with 18β-glycyrrhetinic acid appended pyrene (GA-pyrene, **3**) as the donor, and 2,4,7-trinitrofluorenone (TNF, **4**) as the acceptor. The use of TNF (**4**) as a versatile electron acceptor in the formation of CT gels is demonstrated through the formation of gels in a variety of solvents. Thermal stability, stoichiometry, scanning electron microscopy (SEM), optical micrographs, and circular dichroism (CD) are performed on these CT gels to investigate their thermal and assembly properties. UV–vis, fluorescence, mass spectrometric as well as variable-temperature ^1^H NMR experiments on these gels suggest that the CT interaction is one of the major driving forces for the formation of these organogels.

## Introduction

The creation of one-dimensional (1D) nanostructures is one of the focused fields in supramolecular chemistry [1–4]. In order to control the molecular arrangement in 1D structures, low molecular weight gelators (LMWGs) have generated considerable interest during the past decade [5–6]. The driving forces for the formation of such self-assembled fibrillar networks include hydrogen-bonding interaction, van der Waals force, π–π stacking, and donor–acceptor interaction [7–10]. Since LMWGs are often thermally reversible and the gelation can be triggered by pH or the addition of small molecules (cations, anions), they can potentially be used in biomedicine [11–13], sensing [14–15], optoelectronics [16–17], and other applications [18–19]. Among different LMWGs, the two-component gel has attracted recent attention due to their strength, stability and other properties which can be controlled by varying functionalities and concentrations of each individual component [20–24].

Charge-transfer (CT) interaction exists between π-electron-rich species (donor molecules) and π-electron-deficient species (acceptor molecules), resulting in a characteristic absorption band in the UV–vis region [25]. CT interactions have been employed to induce the supramolecular interactions between donors and acceptors, and lead to the formation of two-component organogels [26–30]. 18β-Glycyrrhetinic acid (GA, **1**), a natural pentacyclic triterpenoid obtained from medicinal plants in the form of free acids or aglycones, has been noted by its low toxicity, biocompatibility and bioactivity [31–32]. A wide array of papers has been published to report its anti-inflammatory, antiviral, and antitumor effects [33–34]. In recent years, it has been found that triterpenoids can serve as powerful building blocks for materials development due to their unique rigid molecular structures [35–41].

In our previous work, we have already reported the synthesis of organogels based on triterpenoid moieties. For example, we designed and studied the one-component organogelation ability of 2,3-dihydroxyiminooleanolic acid, adenineoleanolic acid conjugates as well as the fan-shaped *C*_3_ and molecular tweezers based on glycyrrhetinic acid [42–44]. In this paper, 18β-glycyrrhetinic acid–pyrene (GA-Pyrene, **3**) as the electron donor, and 2,4,7-trinitrofluorenone (TNF, **4**) as the electron acceptor, were synthesized and employed to direct the formation of organogels through the CT interaction.

## Results and Discussion

### Synthesis

**Scheme 1:**
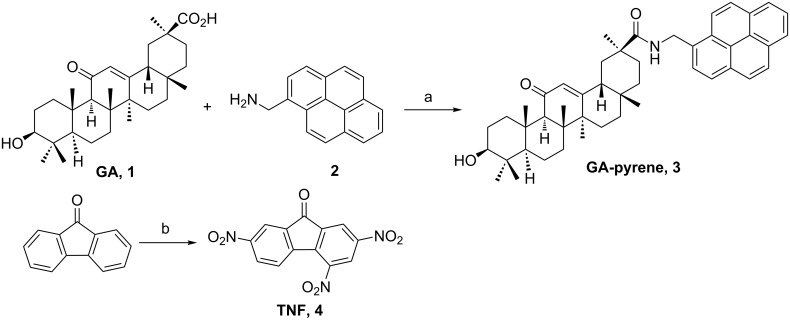
Reagents and reaction conditions: (a) 4-dimethylaminopyridine (DMAP), 1-ethyl-3-(3-dimethylaminopropyl)carbodiimide (EDC), dichloromethane (DCM), room temperature, 72%; (b) HNO_3_, H_2_SO_4_, 70 °C, 93%.

As shown in Scheme 1, 18β-glycyrrhetinic acid reacted with 1-pyrenemethylamine in dichloromethane to give the GA-pyrene conjugate **3** with 72% yield. On the other hand, the 2,4,7-trinitrofluorenone (TNF, **4**) was obtained with 93% yield by nitration of 9-fluorenone.

### Gelation test

**Figure 1:**
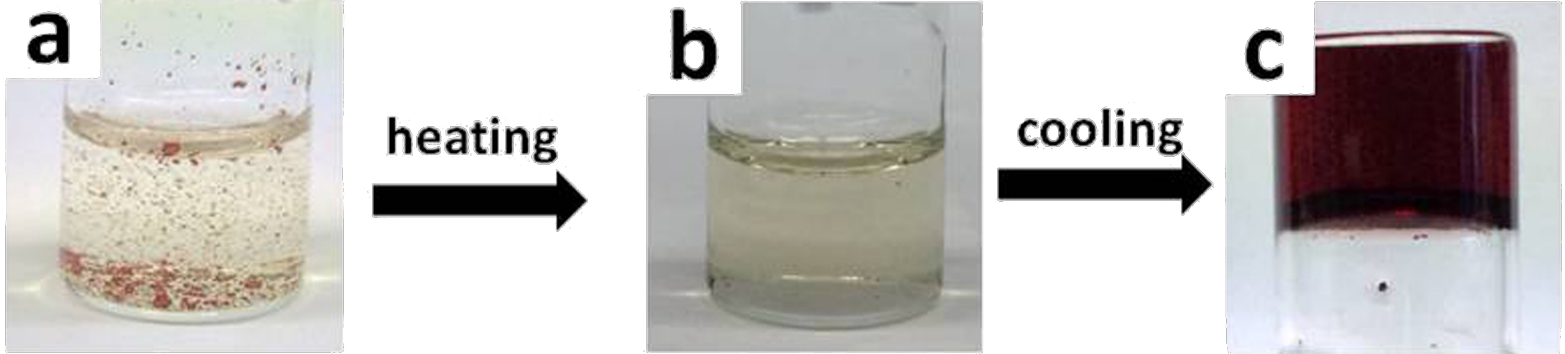
(a) GA-pyrene (**3**) and TNF (**4**, 1:1, molar ratio, [**3**] = 12 mM) in DMSO/water (3:1, v/v). The red insoluble solid is undissolved residue of **3** and **4**. (b) A yellow solution was formed upon heating the mixture at 70 °C. (c) Upon cooling to room temperature, gelation could be observed with intensified red colour.

GA-Pyrene (**3**) was heated in different solvents with 1 equiv of TNF (**4**) until a clear solution was formed. Then, the mixture was allowed to cool down to room temperature, and kept for 30 min to see whether there was any flow of solvent. It showed that dark red CT gels (Figure 1c) were obtained in dimethyl sulfoxide (DMSO)/water and *N*,*N*-dimethylformamide (DMF)/water mixed solvents (Table 1, entries 3, 4 and 9, 10), but not in respective individual solvent system (DMSO, water or DMF, Table 1, entries 1, 6 and 7). At the same time, the ratio between the two solvents also played a key role in the gelation process, as it can be seen in entries 2, 5, 8 and 11 (Table 1). It is probably due to a balance between aggregation and dissolution of the solute molecule in these two solvents [45]. Besides the mixed solvents, gelation and partial gelation also occurred in ethylene glycol (entry 12, Table 1) and acetonitrile (entry 13, Table 1). In aromatic solvents (entries 14 and 15, Table 1), chlorinated solvents (entries 16 and 17, Table 1) and low molecular weight alcohols (entries 18 and 19, Table 1), only precipitates were observed. As the important parameters of stability, the minimum gelator concentration (MGC) and the *T*_gel_ for different CT gels were summarized in Table 1.

**Table 1:** Gelation test results of GA-pyrene (**3**) with TNF (**4**, molar ratio =1:1).

Entry	Solvent	State^a^	MGC^b^	*T*_gel_^c^ (°C)

1	DMSO	S	–	–
2	DMSO/water (7:1, v/v)	S	–	–
3	DMSO/water (5:1, v/v)	G	0.05	46
4	DMSO/water (3:1, v/v)	G	0.03	42
5	DMSO/water (1:1, v/v)	P	–	–
6	water	P	–	–
7	DMF	S	–	–
8	DMF/water (7:1, v/v)	S	–	–
9	DMF/water (5:1, v/v)	G	0.16	39
10	DMF/water (3:1, v/v)	G	0.14	29
11	DMF/water (1:1, v/v)	P	–	–
12	ethylene glycol	G	0.04	62
13	CH_3_CN	PG	0.35	55
14	toluene	P		
15	benzene	P	–	–
16	chloroform	S	–	–
17	methylene chloride	S	–	–
18	*n*-butyl alcohol	P	–	–
19	isopropanol	P	–	–

^a^G = gel, PG = partial gel. S = solution, P = precipitate. ^b^MGC is the minimum gelator concentration (g/100 cm^3^) at which the gel formed. ^c^*T*_gel_ values given here are at the corresponding MGC.

The gelation abilities of **1** with **4**, pyrene-amine **2** with **4**, as well as **3** in the above solvents were also tested as the control experiments. No gel formation was observed in these situations, and a red solution formed for **2** with **4**, indicating the skeletons of both **3** and **4** are necessary.

### Thermochromic phase transition

The CT gels based on GA-pyrene **3** and TNF **4** showed a thermochromic phase transition as all the other organogels [46–47], but the difference in these CT gels showed obvious colour changes from gel to solution state. As shown in Figure 1, a yellow solution around 70 °C transformed to a deep red gel upon cooling to room temperature, and it changed back to the yellow colour when it was heated again. This observation suggested the presence of a CT complex in the gel system.

#### Thermal stability

**Figure 2:**
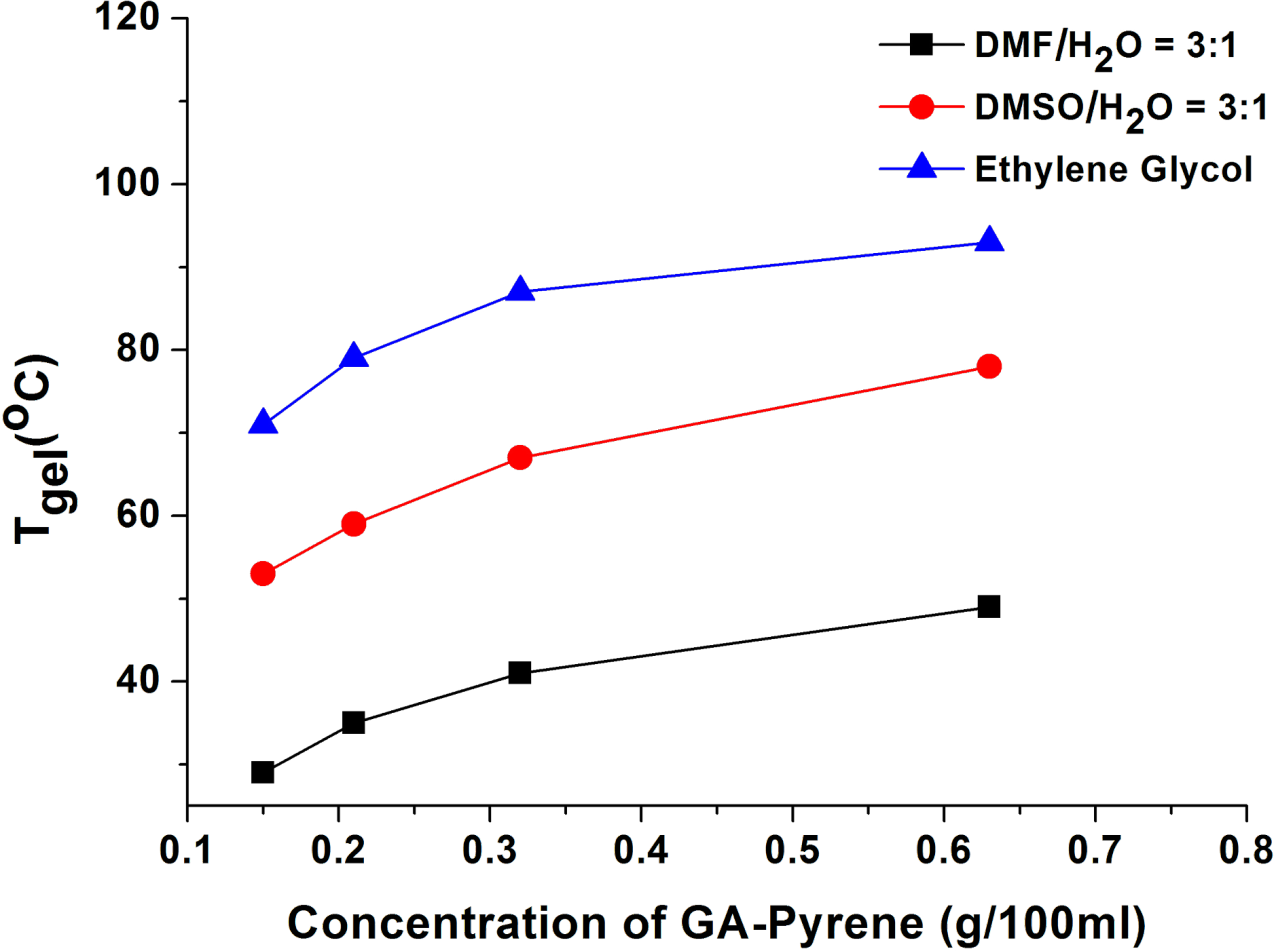
*T*_gel_ of GA-pyrene (**3**) and TNF (**4**, 1:1, molar ratio) with the increasing concentration in different solvents.

Sol to gel transition temperature (*T*_gel_) was measured as a function of the total concentration of the 1:1 (molar ratio) donor–acceptor mixture. These were done by inverted test tube experiments after gels were stabilized in sealed test tubes for 1–2 h [15,48–50]. It was observed that the thermal stability of these CT gels based on **3** and **4** increased with an increase in the total concentration (Figure 2), no matter in what kind of solvent. Based on the *T*_gel_ values at different concentrations, the thermodynamic parameters, i.e. Δ*H*°, Δ*S*°, and Δ*G*° at 298 K in different solvents were calculated and are summarized in Table 2 (for the calculated details see Supporting Information File 1) [51]. The order of Δ*G*° values in different solvents was in accordance with that of their *T*_gel_, and the CT complex of **3** and **4** had the stronger gelation ability in ethylene glycol than in the other solvents.

**Table 2:** Thermodynamic parameters (Δ*H*°, Δ*S*°, and Δ*G*°) of CT gel (**3** and **4**, 1:1, molar ratio) in various solvents at 298 K.

Solvent	Δ*H*° kJ/mol	Δ*S*° J/(mol·K)	Δ*G*° kJ/mol

DMF/water (3:1, v/v)	43.1	36.9	32.1
DMSO/water (3:1, v/v)	51.0	48.5	36.5
ethylene glycol	56.8	57.6	39.6

#### Stoichiometry study

Studies on the thermal stability as a function of stoichiometry were also performed on these CT gels for different molar ratios of GA-pyrene (**3**) and TNF (**4**, 2:1, 3:2, 4:3, 1:1, 3:4, 2:3, 1:2, Figure 3). Interestingly, with increase of the amount of **4** in the CT gels, the *T*_gel_ got the maximum at a 1:1 molar ratio of **3** and **4**. This behaviour was observed in all the three systems, although the maximum of *T*_gel_ is not identical in each system, which was caused by the degree of aggregation of the donor and the acceptor in these CT gels.

**Figure 3:**
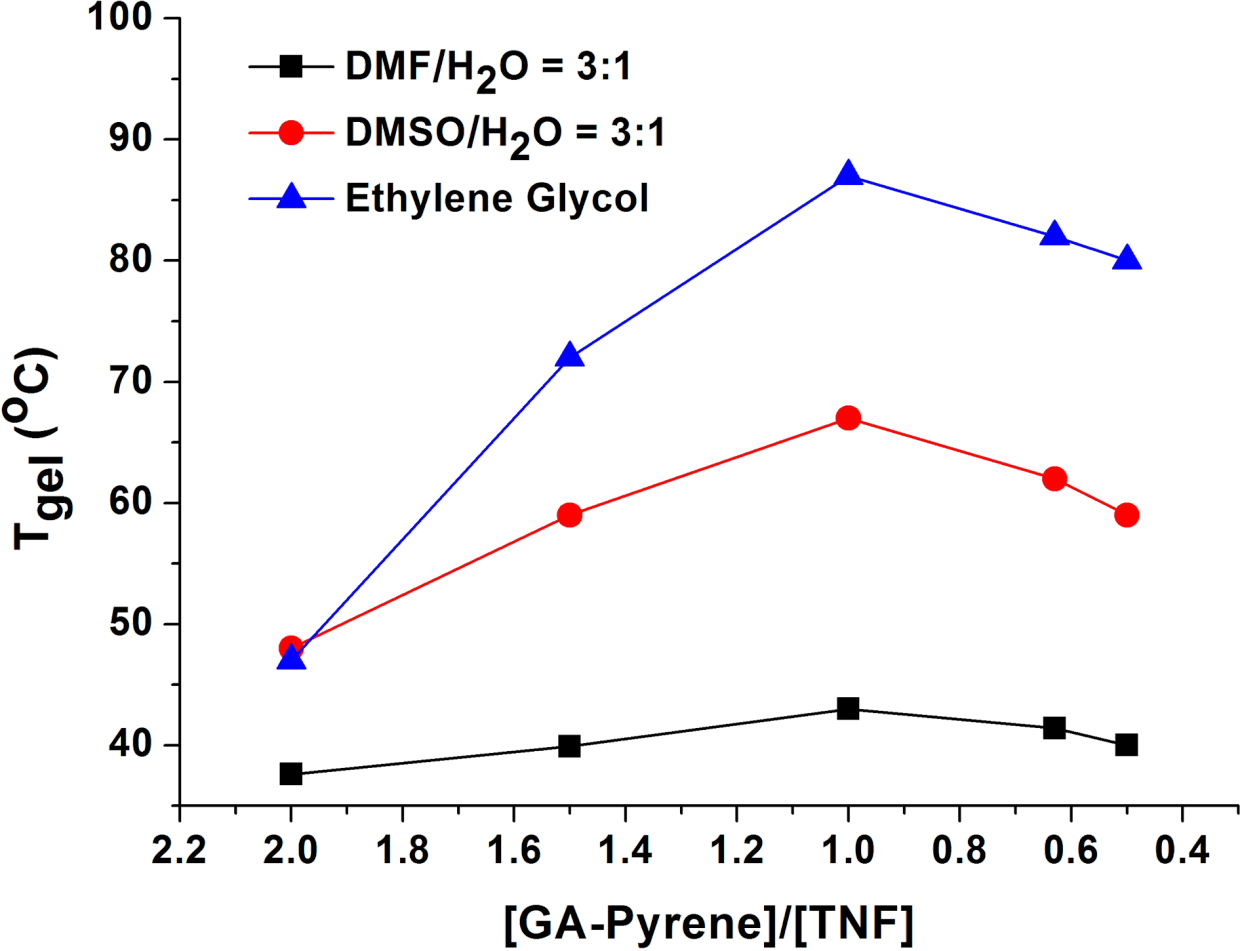
*T*_gel_ of all the CT gels of GA-pyrene (**3**, 0.6 g/100 mL) with TNF (**4**) in varying ratios.

#### Microscopic study

**Figure 4:**
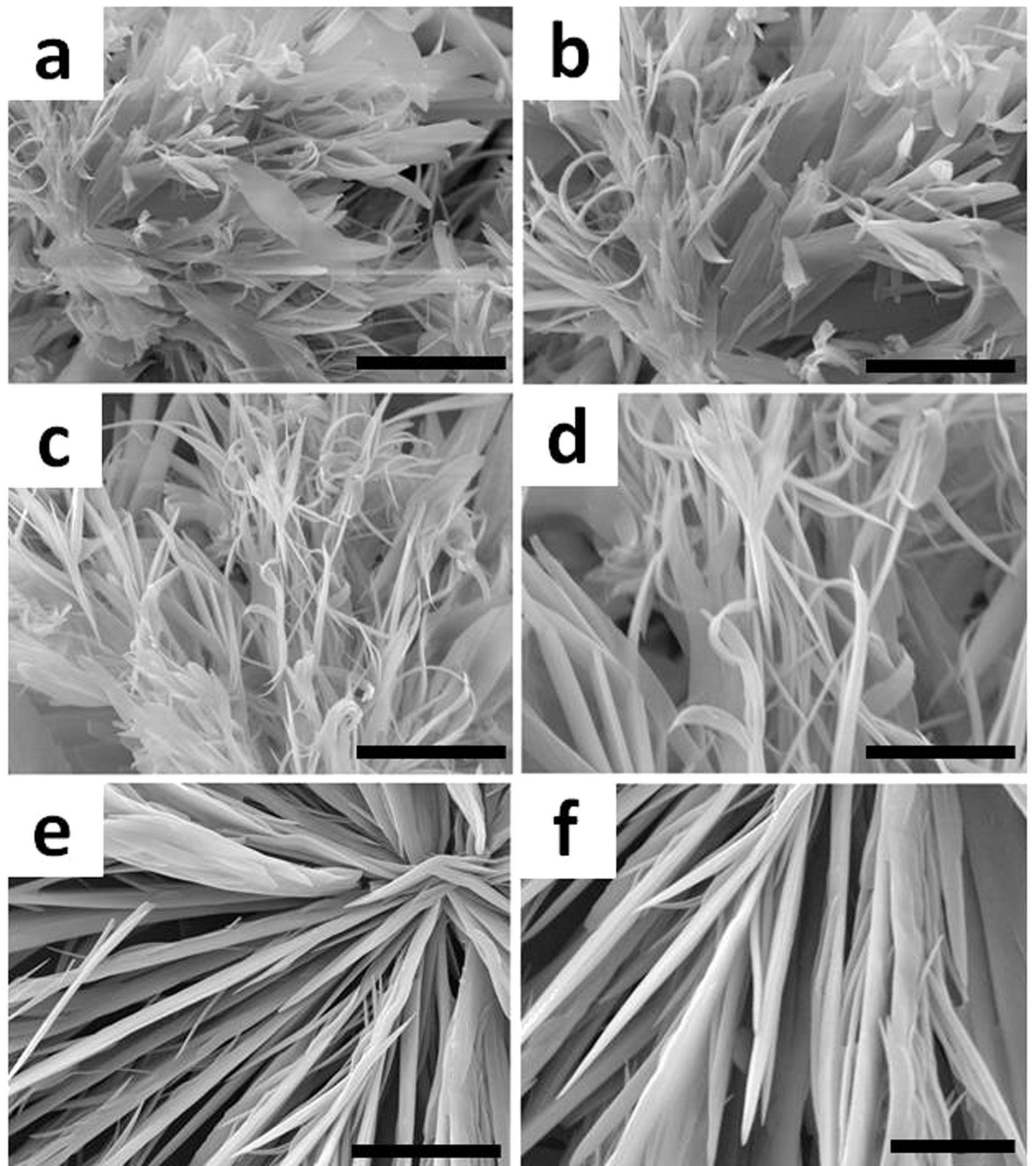
Scanning electron micrographs of CT xerogels (**3** and **4**, 1:1, molar ratio, [**3**] = 9 mM) in DMSO/water (3:1, v/v) (a, b), ethylene glycol (c, d), and DMF/water (3:1, v/v) (e and f). Scale bars in are 20 μm for (a, c, e) and 10 μm for (b, d, f).

**Figure 5:**
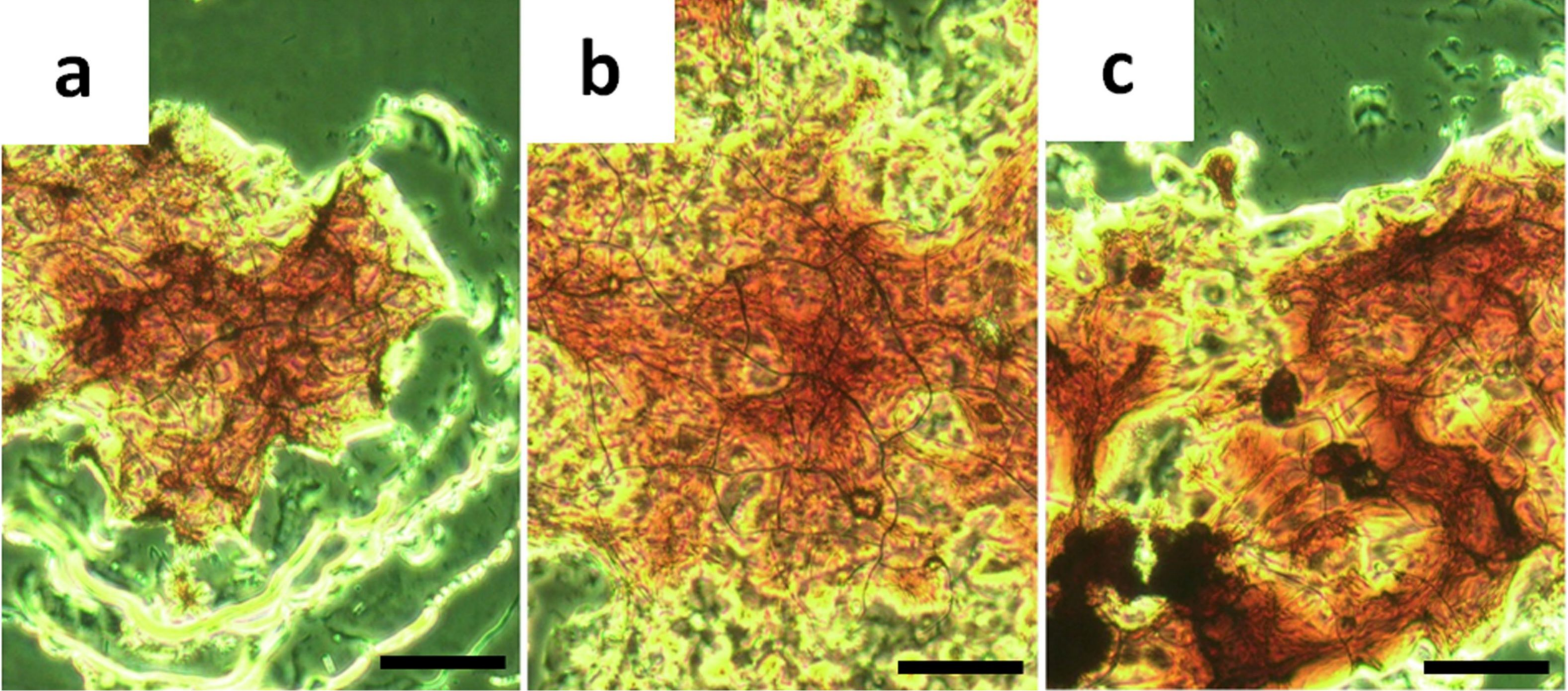
Optical micrographs (20 × 20) of CT xerogels (**3** and **4**, 1:1, molar ratio, [**3**] = 9 mM) in (a) DMSO/water (3:1, v/v), (b) ethylene glycol, and (c) DMF/water (3:1, v/v). Scale bar is in 0.1 mm.

Generally, it is believed that the gelation system is the result of a gelator network formed during the cooling or shaking process [52]. The morphology of the xerogels obtained from CT gels was investigated by scanning electron microscopy (SEM) in order to measure these kinds of networks or gelator aggregates. As shown in Figure 4, the CT xerogel based on GA-pyrene (**3**) and TNF (**4**) in different solvents could self-assemble into micro-scale fibrous structures with regular diameters of ca. 0.5–3 μm, and by entanglement of such fibers, a closely packed 3D network structure was created to trap the solvent molecules into its interstices. The fiber sizes of these two-component CT gels were larger than those of one-component organogels obtained by us previously [42–44]. On the other hand, these xerogels also revealed various types of morphologies based on the different solvent systems. Figure 4e and 4f show that the xerogels formed in a DMF/water system are made of more rigid and larger fibers than those of the other gels under the same concentration. Because of the large size of fibers, optical microscopy was also used to investigate their aggregates. As shown in Figure 5, the fiber aggregates can be observed clearly with the red colour which can be attributed to the charge transfer band [53–54].

#### Chirality study

**Figure 6:**
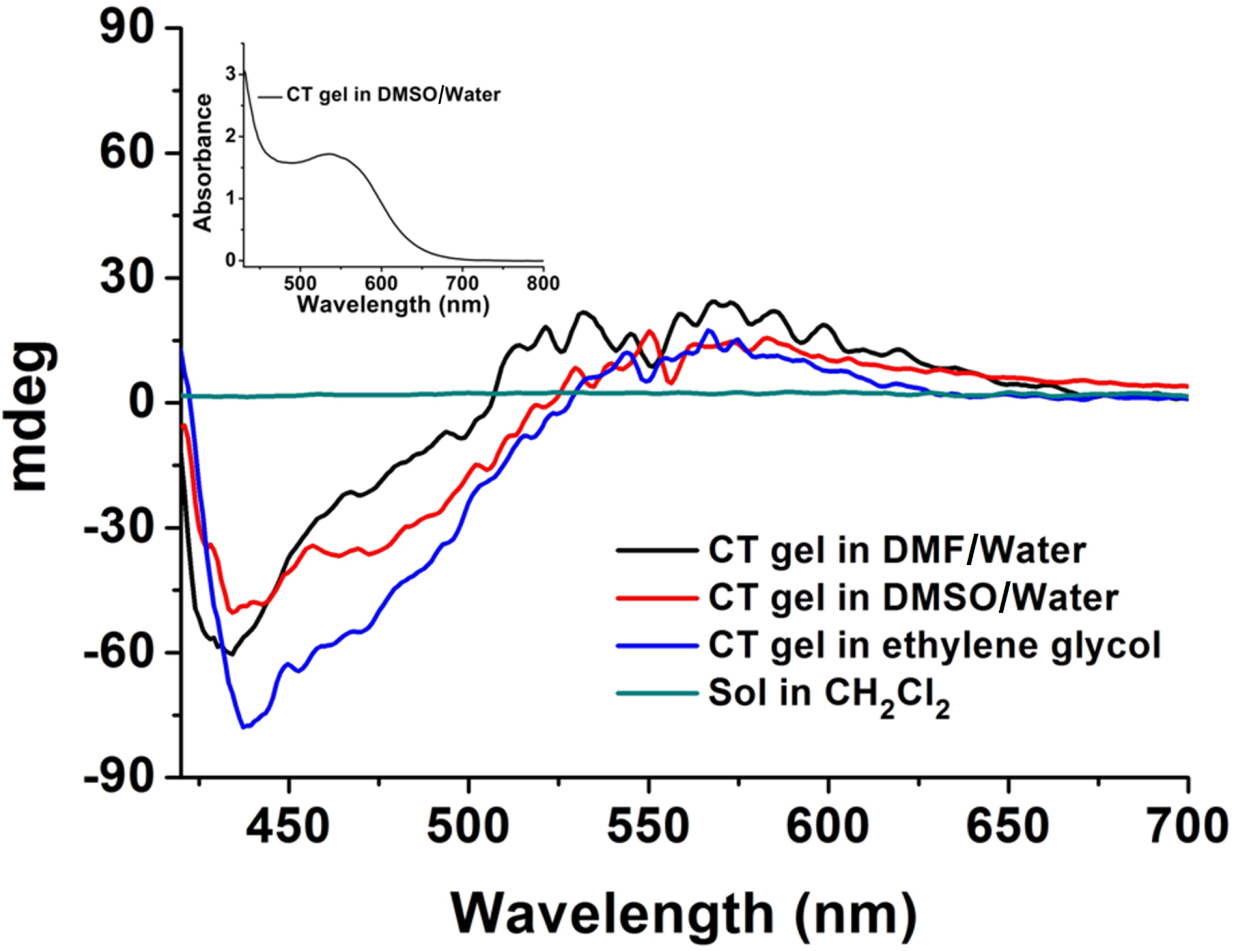
CD spectra of sol in CH_2_Cl_2_, and CT gel obtained from DMF/water (3:1, v/v), DMSO/water (3:1, v/v), and ethylene glycol (**3** and **4**, 1:1, molar ratio, [**3**] = 12 mM); inset graph: UV–vis spectrum of CT gel in DMSO/water (3:1, v/v).

It is known that many chiral gelators can form helical or twisted aggregates upon gelation [55–56]. Even achiral gelators have been reported to aggregate in both forms, right-handed and left-handed helices in equal proportions [57–58]. Therefore, circular dichroism (CD) was used to investigate the chirality of these CT gels and sol. The results showed a strong negative signal around 430 nm and a positive signal around 550 nm contrasted with the CD silence of the sol (Figure 6), which indicated these CT gels were indeed chiral aggregates [59–61].

#### Charge-transfer interaction

**Figure 7:**
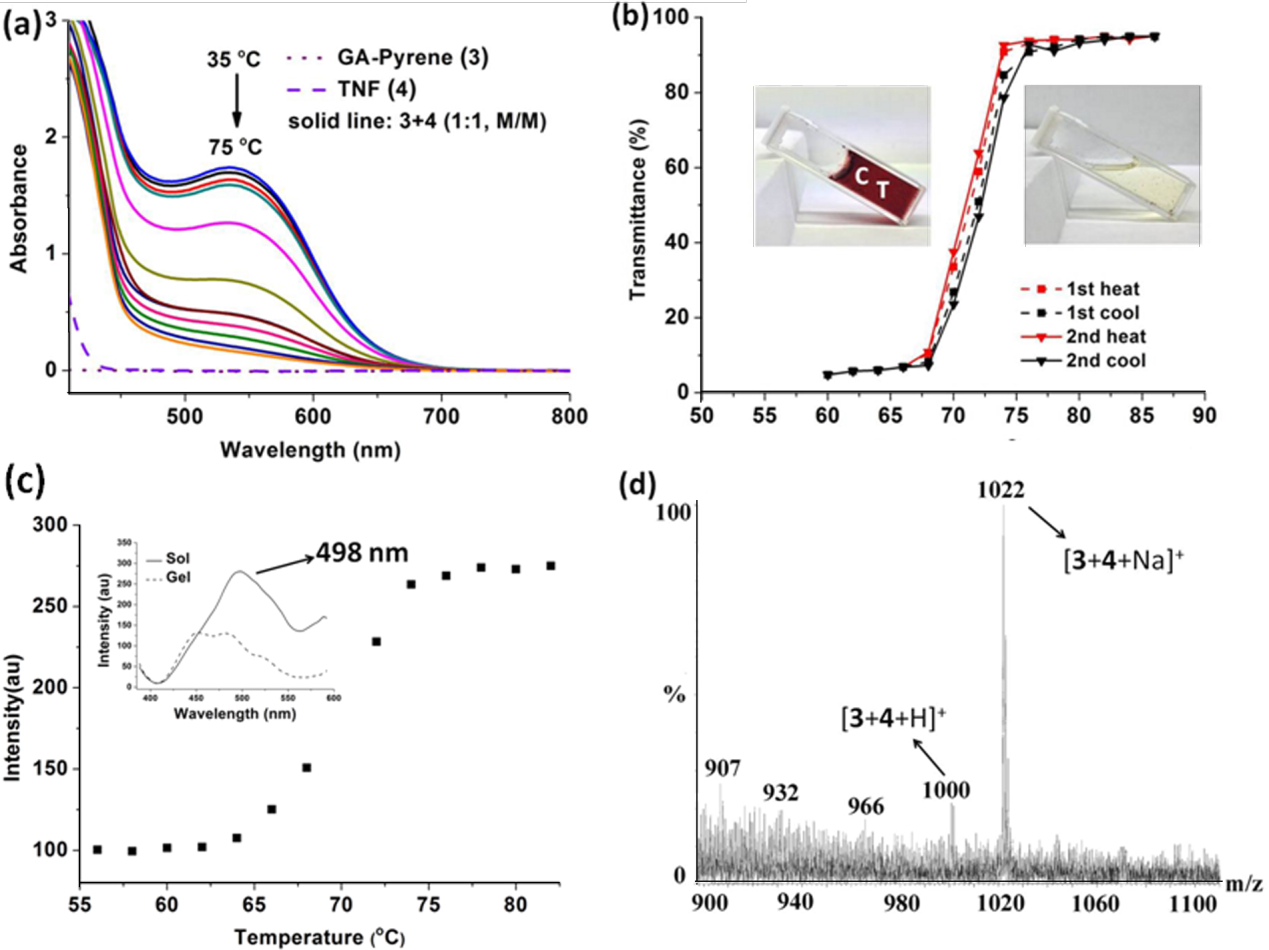
(a) UV–vis, (b) transmittance at 700 nm, (c) fluorescence intensity at 498 nm (excitation: 365 nm) at various temperatures of CT gel (**3**/**4** = 1:1, molar ratio) in DMSO/water (3:1, v/v), inset: fluorescence spectra of gel and sol, [**3**] = 12 mM], and (d) ESIMS (+) of **3** and **4** (1:1, molar ratio) in acetonitrile.

For these gel systems, the CT between the donor and acceptor molecules is believed to be one of the major driving forces. The CT interaction between **3** and **4** (1:1, molar ratio) in DMSO/water (3:1, v/v) was studied by UV–vis spectroscopy (Figure 7a). The absorbance at 547 nm (CT absorption band) decreased with increasing temperature (35 °C to 75 °C), and the colour changed from dark red to light yellow (inset of Figure 7b), indicating the dissociation of CT complexes [62–63]. Meanwhile, the transmittance changes of the CT gel in DMSO/water (3:1, v/v) at different temperatures are given in Figure 7b. A drastic change was observed upon heating to ~73 °C, and after cooling to room temperature, the second heating gave the almost same results. The formation of the CT complex was further confirmed by fluorescence spectroscopy. The emission intensity of the CT gel at 498 nm (excitation wave length: 365 nm) above 75 °C is almost two times stronger than the one below 65 °C, and the largest changes were observed from 68 °C to 75 °C (Figure 7c), which is consistent with the absorbance and transmittance spectra. Furthermore, there was a blue shift between the CT gel and sol status (inset of Figure 7c), which may be due to the decrease of intermolecular charge transfer in the sol status compared to the gel state [24]. Finally, the molecular ion at *m*/*z* 1000 [**3** + **4** + H]^+^ and 1022 [**3** + **4** + Na]^+^ in Figure 7d afforded a direct evidence to indicate the strong CT interaction between **3** and **4** (1:1, molar ratio).

#### Variable-temperature ^1^H NMR study

**Figure 8:**
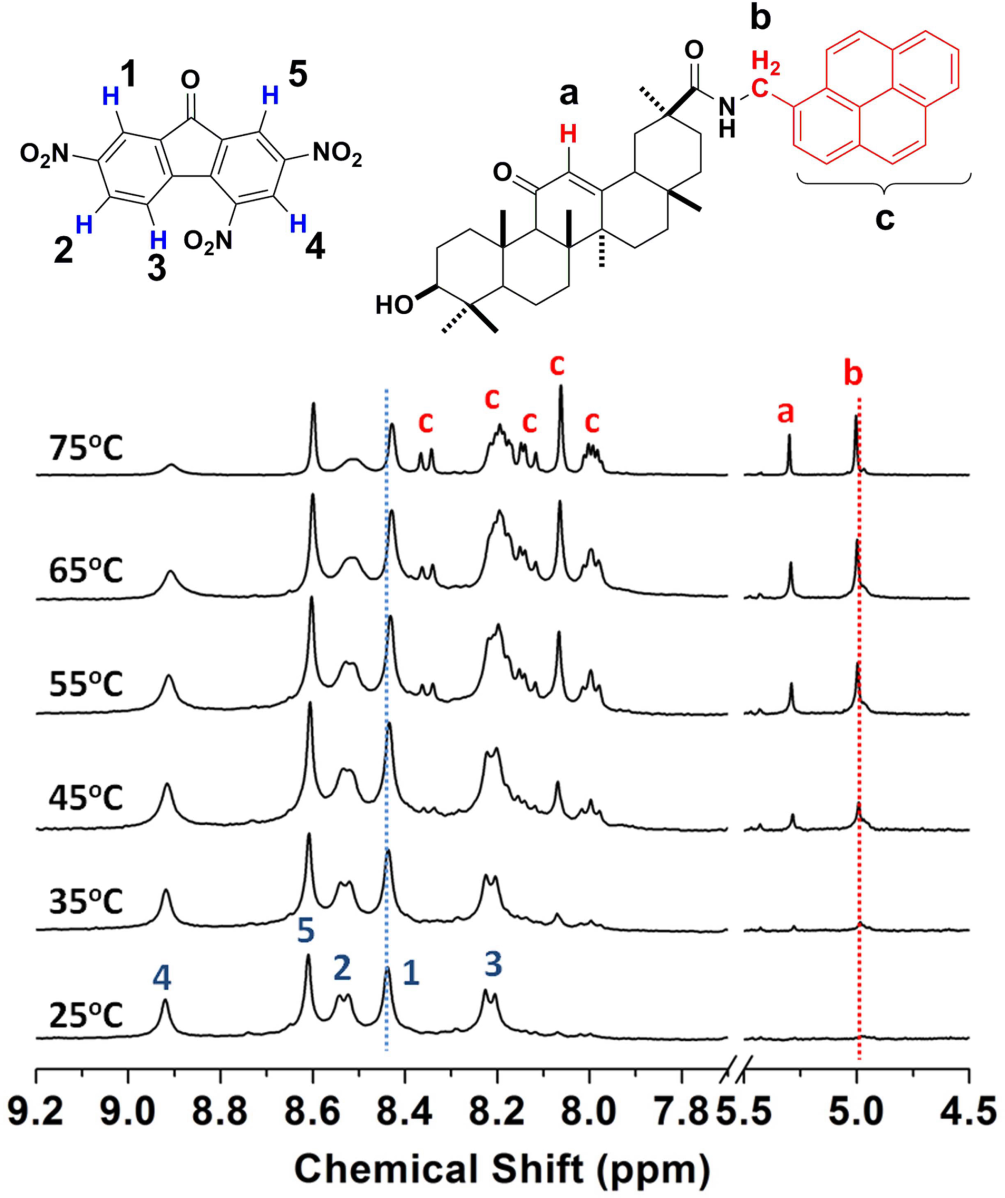
Variable temperature ^1^H NMR (400 MHz) of CT gel (**3** and **4**, 1:1, molar ratio) in DMSO/D_2_O (3:1, v/v). [**3**] = 9 mM.

Since the changes of driving force intensity can lead to a difference of chemical shifts in the ^1^H NMR spectra, a variable-temperature ^1^H NMR experiment was carried out of the CT gel in DMSO/D_2_O (3:1, v/v). From 25 °C to 75 °C, data from six groups of protons was collected and is shown in Figure 8. In the gel status (25 °C), almost no proton signals of **3** could be observed, but the signals became sharper and sharper with increasing temperature for both aromatic protons and aliphatic protons. Moreover, the chemical shift of protons from TNF (**4**) shifted slightly upfield, and the double peaks (blue H2 and H3 in Figure 8) became broader till they merged into one single peak, which can be attributed to the change of the charge transfer interaction between **3** and **4**, and the increased *T*_2_ relaxation rate at higher temperature driven by scalar coupling to the adjacent nitro group [64]. The high temperature made the donor and the acceptor more mobile, resulting in the decrease of the CT interactions.

## Conclusion

A two-component CT interaction induced an organogel formation based on 18β-glycyrrhetinic acid–pyrene conjugate **3** and 2,4,7-trinitrofluorenone (TNF, **4**). Their thermal and assembly properties were studied by thermal stability, stoichiometry, scanning electron microscopy (SEM), optical micrographs, and circular dichroism (CD) experiments. Meanwhile, the results of UV–vis, fluorescence, mass spectrometry, as well as variable-temperature ^1^H NMR experiments on these gels suggested that the charge-transfer interaction is the major driving force for the formation of these CT gels.

## Experimental

### Materials and measurement method

1-Pyrenemethylamine hydrochloride (95%), 18β-glycyrrhetinic acid (GA, 97%), 4-dimethylaminopyridine (DMAP, 99%), 1-ethyl-3-(3-dimethylaminopropyl)carbodiimide (EDC, 99%), 9-fluorenone (98%) are purchased from Sigma-Aldrich, and used as received. All organic solvents were dried and distilled before used.

UV–vis spectra were measured on an Agilent Technologies 95-03 spectrometer; fluorescence spectra were measured on a Varian Cary Eclipse spectrometer; NMR spectra were recorded on Varian Mercury 300/400 spectrometers in CDCl_3_ and DMSO-*d*_6_; mass spectrometry was measured on a Micromass QTOF and Finnigan TSQ spectrometer in positive mode; circular dichroism spectra were performed on JASCO J-815 CD spectrometer; scanning electron microscopy (SEM) images were performed on a Variable Pressure Tescan Vega3 SBU; optical micrographs were performed on a Olympus CKX41 microscope.

Samples for optical micrographs are prepared by cutting a piece of gel and putting it on the surface of a glass plate to dry at room temperature. Samples for SEM are prepared by flash freezing in liquid nitrogen followed by lyophilisation, and the freeze-dried sample was placed on a silica surface to be coated by gold before the SEM test. The minimum gelation concentration (MGC) was measured by a weighted amount of gelator and an increasing volume of solvent in a 4 mL glass vial. The gel-to-sol transition temperatures (*T*_gel_) were determined by an “inverted test tube” method [15].

### Synthesis of 18β-glycyrrhetinic acid appended pyrene **3**

To a solution of 1-pyrenemethylamine hydrochloride (113 mg, 0.42 mmol), 4-dimethylaminopyridine (DMAP, 52 mg, 0.42 mmol) and 1-ethyl-3-(3-dimethylaminopropyl)carbodiimide (EDC, 135 mg, 0.42 mmol) in dry dichloromethane (DCM, 30 mL), 18β-glycyrrhetinic acid (GA, 200 mg, 0.42 mmol) was added during 2 h. After the addition, the reaction solution was stirred for 5 h at room temperature, and then washed with water and brine. The organic layer was combined, dried with sodium sulfate and filtered, followed by the removal of solvents under reduced pressure. The crude product was purified by column chromatography (ethyl acetate/hexane = 1:1 v/v) on silica to give pure compound **3** as a yellow powder, 208 mg, 72% yield. ESIMS (+) *m*/*z*: 684 [M + H]^+^, 701 [M + NH_4_]^+^; mp 215–216 °C; HRMS (ESI): *m*/*z* [M + H]^+^ calcd for C_47_H_58_NO_3_, 684.4414; found, 684.4416; ^1^H NMR (300 MHz, CDCl_3_) 8.11–7.81 (m, 9H, pyrene-H), 6.33 (m, 1H, NHCO), 5.46 (s, 1H, 12-CH), 5.22 (dd, *J*_1_ = 12 Hz, *J*_2_ = 3 Hz, 1H, NCH_2_-pyrene), 5.00 (dd, *J*_1_ = 12 Hz, *J*_2_ = 3 Hz, 1H, NCH_2_-pyrene), 3.11 (t, *J* = 7 Hz, 1H, 3-H), 2.66 (m, 1H, 3-OH), 2.17–0.77 (m, 21H, CH_2_ and CH), 0.68, 0.74, 0.87, 0.92, 1.10, 1.15, 1.24 (7×s, 7×3H, 23, 24, 25, 26, 27, 28, 29-CH_3_); ^13^C NMR (75 MHz, CDCl_3_) 199.85 (11-C), 175.36 (30-CONH), 169.03 (13-C), 131.49, 131.20, 130.75, 128.97, 128.45, 128.21, 127.48, 127.33, 127.25, 126.04, 125.33, 125.00, 124.68, 123.01 (pyrene-C, 12-C), 78.74 (3-C), 61.65, 54.09, 48.14, 45.22, 43.76, 43.06, 42.05, 41.42, 39.04, 37.45, 36.88, 32.62, 31.86, 31.41, 29.42, 28.31, 27.14, 26.35, 23.34, 23.18, 18.38, 17.39, 16.21, 16.05, 15.53, 15.40.

### Synthesis of 2,4,7-trinitrofluorenone (**4**)

The synthesis of **4** was carried out by the reported method [65]. The purity of **4** was verified by NMR spectroscopy, thin-layer chromatography, mass spectrometry and melting point (mp 170–172 °C, ref. [65] mp 169–171 °C). All the experimental data of the isolated product was coincident with those previously reported [65]. EIMS (+) *m*/*z*: 315; ^1^H NMR (300 MHz, DMSO-*d*_6_) 8.98 (d, *J* = 2 Hz, 1H, 3-H), 8.42 (d, *J* = 2 Hz, 1H, 1-H), 8.18 (d, *J* = 8 Hz, 1H, 5-H), 8.61 (m, 2H, 6-H, 7-H); ^13^C NMR (75 MHz, DMSO-*d*_6_) 186.41, 149.67, 148.89, 144.80, 143.34, 138.85, 137.85, 136.16, 130.78, 128.00, 125.98, 122.19, 118.99.

## Supporting Information

File 1 MS, ^1^H NMR and ^13^C NMR spectra of 18β-glycyrrhetinic acid appended pyrene **3** and 2,4,7-trinitrofluorenone (**4**); thermodynamic parameters of CT gel in various solvents.
