# Supplementary material for: Charge-transfer interaction mediated organogels from 18β-glycyrrhetinic acid appended pyrene
Source: Beilstein J Org Chem. 2013 Dec 16;9:2877–85. doi: 10.3762/bjoc.9.324 (PMC3869347; doi:10.3762/bjoc.9.324)
Supplement: File 1 — MS, 1H NMR and 13C NMR spectra of 18β-glycyrrhetinic acid appended pyrene 3 and 2,4,7-trinitrofluorenone (4); thermodynamic parameters of CT gel in various solvents. [file Beilstein_J_Org_Chem-09-2877-s001.pdf]

Charge-transfer interaction mediated organogels from 18β-glycyrrhetinic acid appended pyrene

Jun Hu,[1, 2] Jindan Wu,[1] Qian Wang*[2] and Yong Ju*[1]

Address: [1]Key Laboratory of Bioorganic Phosphorus Chemistry & Chemical Biology, Ministry of Education, Department of Chemistry, Tsinghua University, Beijing, 100084, China, and [2]Department of Chemistry and Biochemistry, University of South Carolina, Columbia, 29208, USA.

Email: Yong Ju* - juyong@tsinghua.edu.cn; Qian Wang* - WANG263@mailbox.sc.edu

*Corresponding authors

MS, ^{1}H NMR and ^{13}C NMR spectra of 18β-glycyrrhetinic acid appended pyrene 3 and 2,4,7-trinitrofluorenone (4); thermodynamic parameters of CT gel in various solvents

Content

1. MS, ^{1}H NMR and ^{13}C NMR spectra of 18β-glycyrrhetinic acid appended pyrene **3**……………………………………………………………………………………………s3
2. MS, $^{1}HNMR$ and ^{13}C NMR spectra of 2,4,7-trinitrofluorenone (**4**)…………………………………………………………………………………………………….s5
3. Thermodynamic parameters of CT gel in various solvents……………………s7
4. References……………………………………………………………………………………s9

1. MS, ^{1}H NMR and ^{13}C NMR spectra of 18β-glycyrrhetinic acid appended pyrene (3)

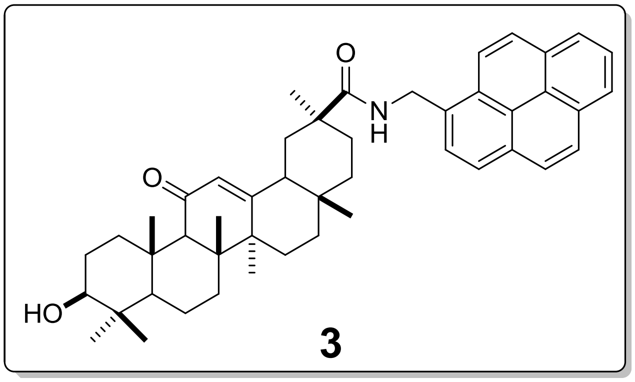

3

ESI-MS (+) Spectra of 18β-glycyrrhetinic acid appended pyrene **3**

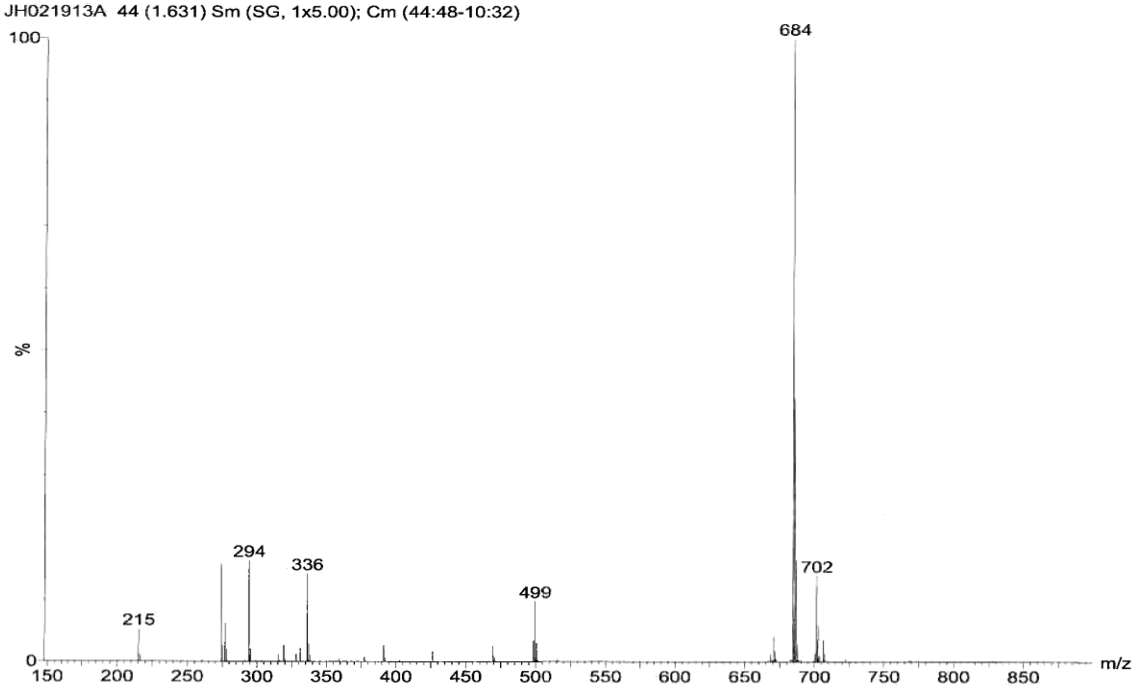

^{1}H NMR Spectra ($CDCl_3$, 300 MHz) of 18β-glycyrrhetinic acid appended pyrene **3**

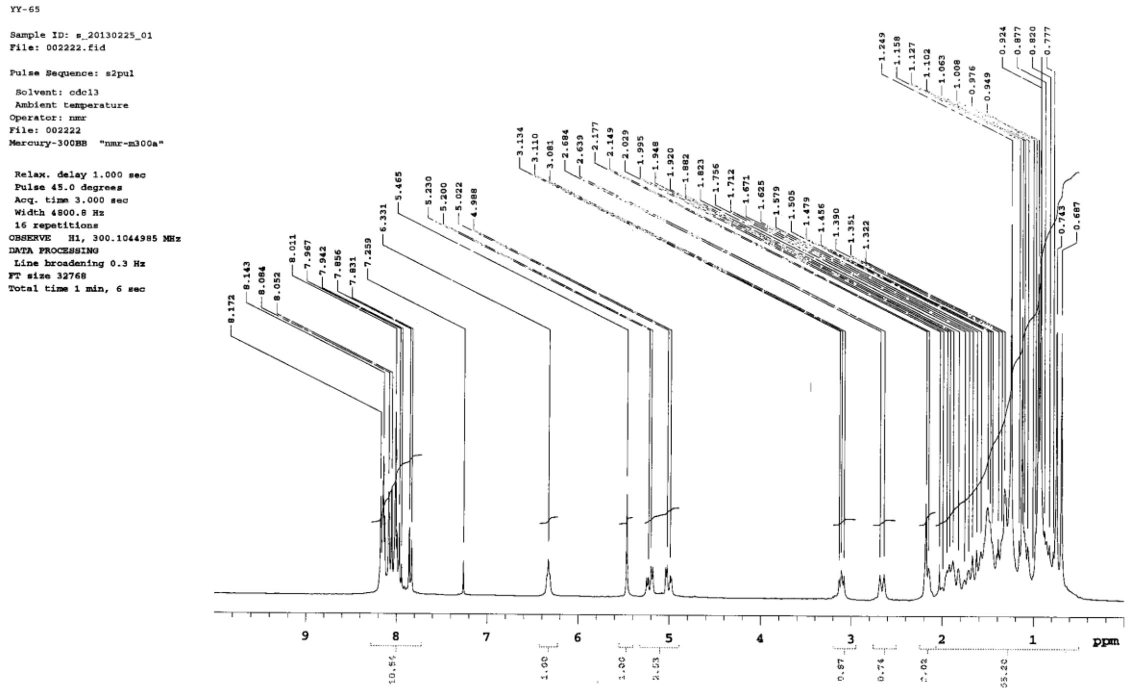

^{13}C NMR Spectra ($CDCl_3$, 75 MHz) of 18β-glycyrrhetinic acid appended pyrene **3**

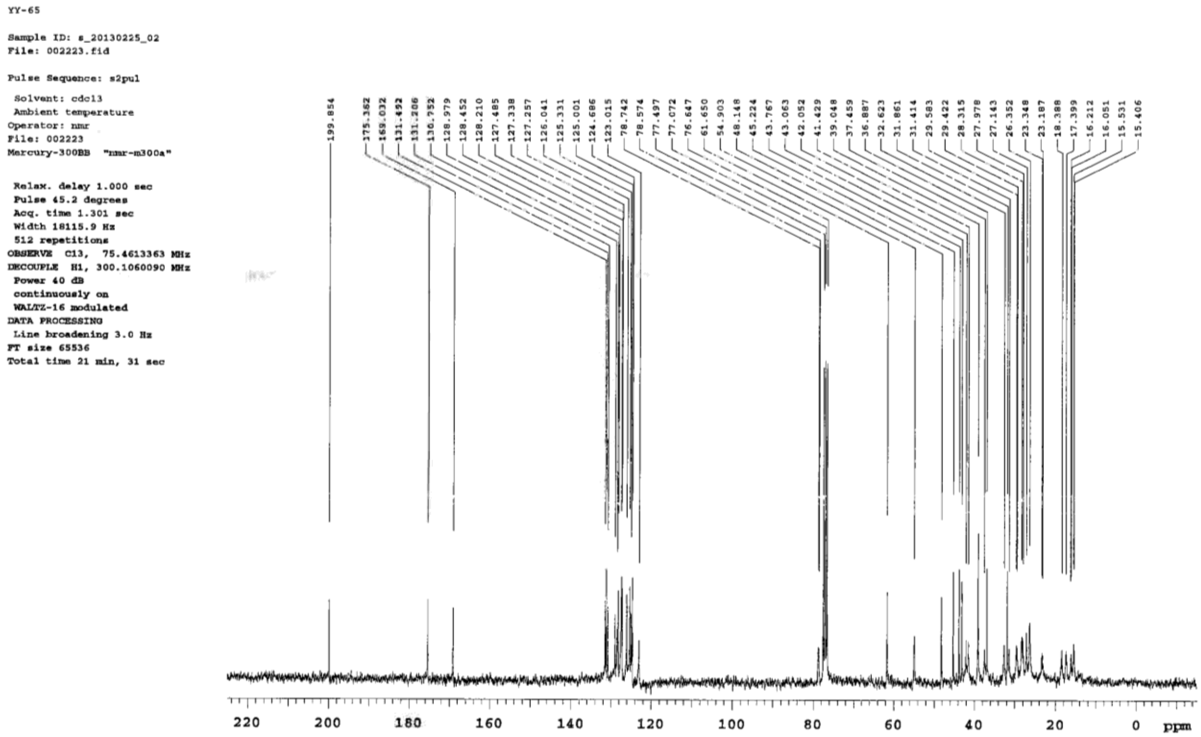

2. MS, ^{1}H NMR and ^{13}C NMR spectra of 2,4,7- trinitrofluorenone (4)

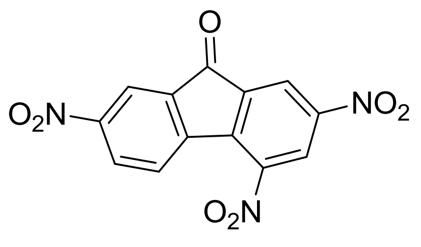

4

EI-MS (+) Spectra of 2, 4, 7-trinitrofluorenone (**4**)

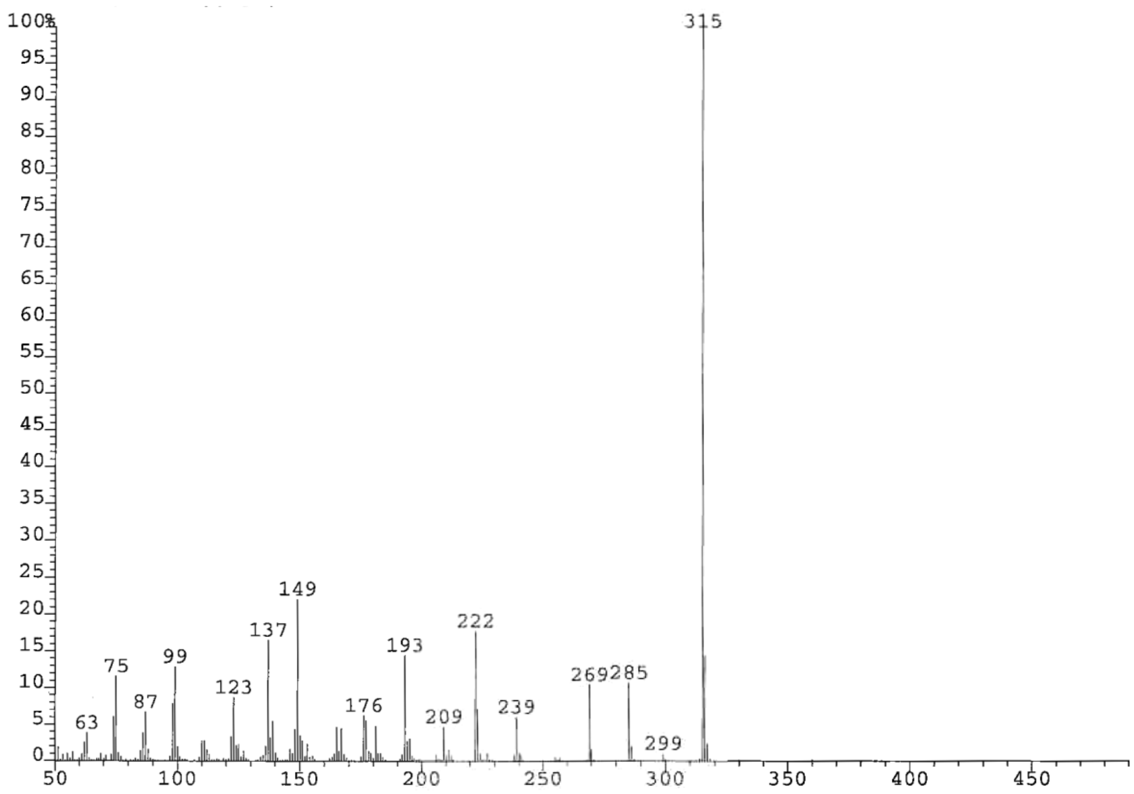

^{1}H NMR Spectra (DMSO, 300 MHz) of 2, 4, 7-trinitrofluorenone (**4**)

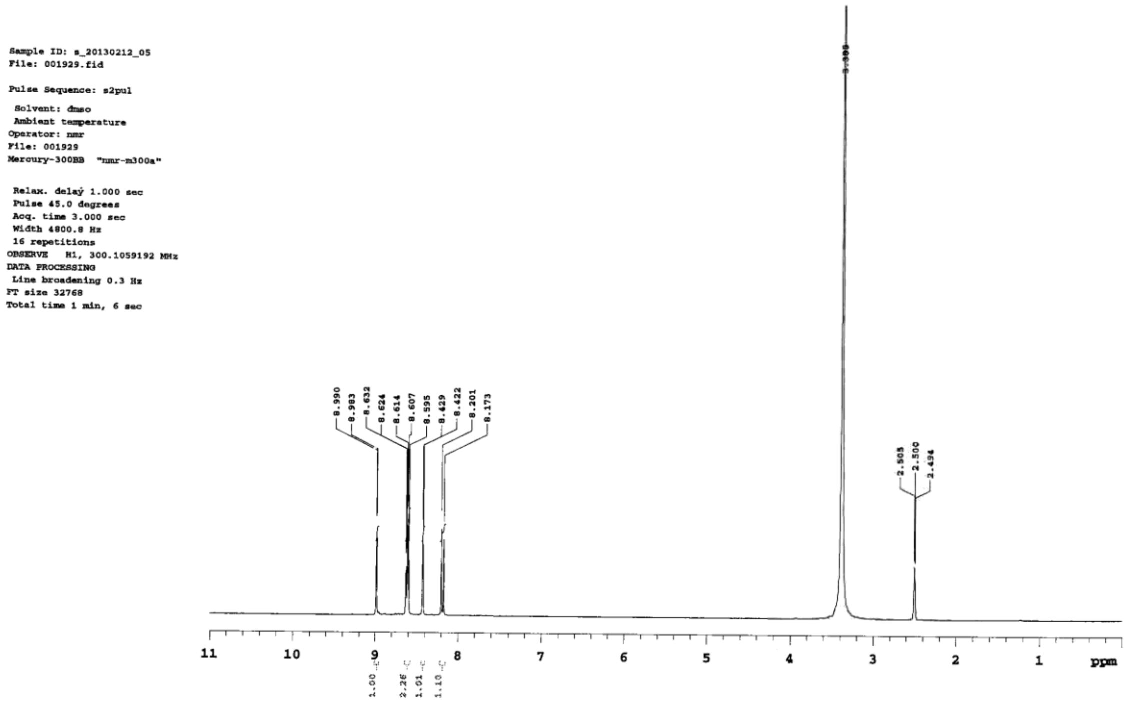

^{13}C NMR Spectra (DMSO, 75 MHz) of 2, 4, 7-trinitrofluorenone (**4**)

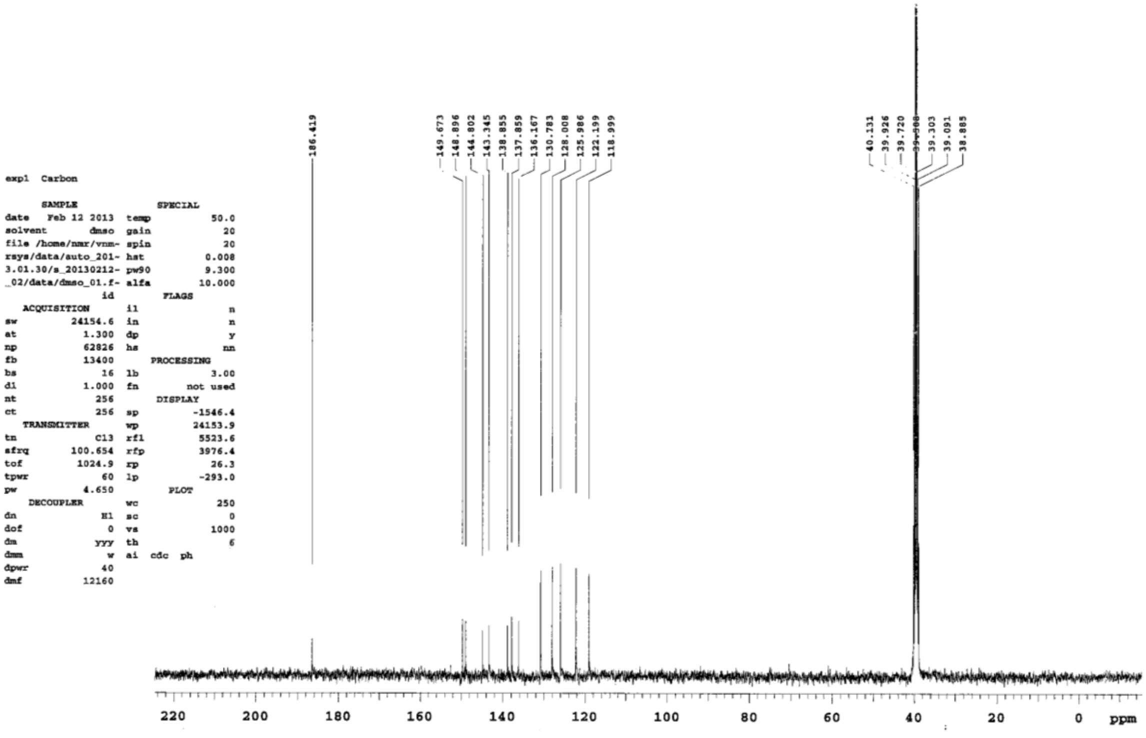

3. Thermodynamic parameters of CT gel in various solvents [1,2]

The thermoreversible melting of a two component gel can be expressed as:

Gel $\rightleftarrows$ liquid

For one component gel, the equilibrium constant can be expressed as:

K = [Gelator] / [Gel]

Assuming unit activity of the gel and taking the concentration of the solution to be equal to the dissolved concentration of the gelator, the equilibrium constant can be expressed as:

K = [Gelator].

The Gibbs free energy change during gel melting can be expressed as:

$\Delta G^o = -\Delta RT \ln K = \Delta H^o - T\Delta S^o$,

Hence, $\ln K = -\Delta H^o / R\ (1/T) + T\Delta S^o / R$

The gel melting temperature (T_{gel}) increases with the concentration of the "solutes". A plot of ln$K$ vs 1/T allowed us to calculate the thermodynamic parameters.

DMF/H_2O (3:1, v/v)

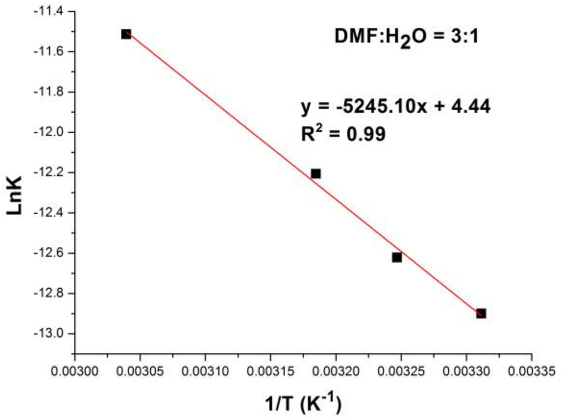

$\ln K = -5245.10 \times (1/T) + 4.44$, $R^2 = 0.99$

$\Delta H^o/R = 5245.10$, ΔH^o = 43.1 kJ/mol;

$\Delta S^o/R = 4.44$, ΔS^o = 36.9 J/mol/K

$\Delta G^o = \Delta H^o - T\Delta S^o = 43.1 - 298 \times 0.0369 = 32.1$ kJ/mol

DMSO/H_2O (3:1, v/v)

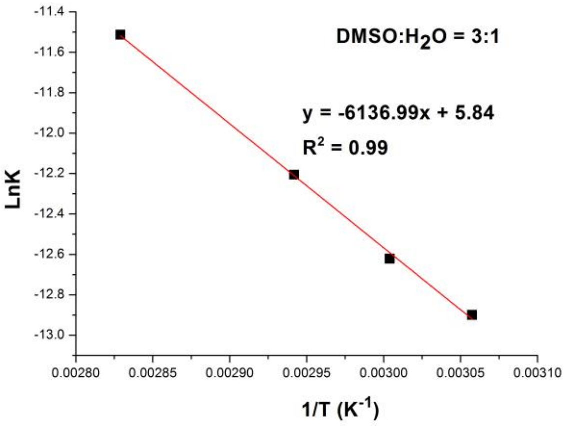

LnK = −6136.99 × (1/T) + 5.84, R^2 = 0.99

$\Delta H^o/R$ = 6136.99, ΔH^o = 51.0 kJ/mol;

$\Delta S^o/R$=5.84, ΔS^o = 48.5 J/mol/K

ΔG^o=ΔH^o − $T\Delta S^o$ = 51.0 – 298 × 0.0485 = 36.5 kJ/mol

Ethylene Glycol

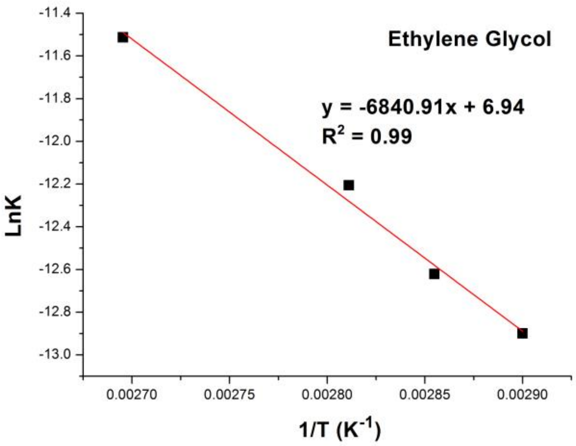

LnK = −6840.91 × (1/T) + 6.94, R^2 = 0.99

$\Delta H^o/R$ = 6840.91, ΔH^o = 56.8 kJ/mol;

$\Delta S^o/R$ = 6.94, ΔS^o = 57.6J/mol/K

ΔG^o = ΔH^o − $T\Delta S^o$ = 56.8 – 298 × 0.0576 = 39.6 kJ/mol

References

1 D. Rizkov, J. Gun, O. Lev, R. Sicsic and A. Melman, *Langmuir,* **2005,** *21,* 12130.

2 J. Hu, M. Zhang and Y. Ju, *Soft Matter*, **2009**, *5*, 4971.
